# The Relationship between TTF-1 Expression and EGFR Mutations in Lung Adenocarcinomas

**DOI:** 10.1371/journal.pone.0095479

**Published:** 2014-04-17

**Authors:** Wan Shanzhi, Han Yiping, Huang Ling, Zheng Jianming, Li Qiang

**Affiliations:** 1 The Respiratory Department, Shanghai Changhai Hospital, SMMU, Shanghai, China; 2 The pathology department, Shanghai Changhai Hospital, SMMU, Shanghai, China; University of North Carolina School of Medicine, United States of America

## Abstract

**Objective:**

To explore the relationship between TTF-1 and EGFR mutations in lung adenocarcinoma tissues to guide clinical treatment timely and effectively.

**Materials and Methods:**

we collected 664 tissue samples from patients with histologically confirmed lung adenocarcinoma from May 2010 to April 2013. All tumor tissues were collected prior to administering therapy. TTF-1 was detected byimmunohistochemistry and EGFR mutations by DNA direct sequencing. Finally, the correlation between TTF-1 expression and the presence of EGFR mutations was analyzed using χ^2^ test or Fisher’s exact test with SPSS software version 18.0.

**Results:**

Of the 664 lung adenocarcinoma tissue samples, 18 were partially positive for TTF-1 (+−), and 636 were positive for TTF-1 (+) resulting in a total positive rate of 98.49% (+,+−)(including partial positive). In only 10 cases was the TTF-1 negative (−); the negative rate was 1.51%. There were 402 cases without an EGFR mutation and 262 cases with EGFR mutations; the rate of mutations was 39.46%. The location of the EGFR mutation was exon 19 for 121 cases resulting in a mutation rate in exon 19 of 18.22%. The location of the EGFR mutation was exon 21 for 141 cases resulting in a mutation rate in exon 21 of 21.23%. Exon 18 and 20 detected by DNA direct sequencing no mutations.A Fisher’s exact test was used to determine the correlation between EGFR mutations and TTF-1 expression.for the whole, TTF-1 positive expression(including partial positive) has correlation with EGFR mutations (p<0.001),especially for Exon 21 expression,the correlation is significant (p = 0.008).

**Conclusion:**

In lung adenocarcinomas, positive and partial positive TTF-1 expression has a significant positive correlation with EGFR mutations(exon 19 and 21). In clinical practice, TTF-1 expression combine with EGFR mutations, especially exon 21 mutation can guide clinical treatment timely for lung adenocarcinomas.

## Introduction

Lung cancer is one of the leading causes of cancer death [1]the five-year survival rate of lung cancer patients is about 15% [2]. Non-small cell lung cancer accounts for approximately 80% of lung cancers, and adenocarcinoma is one of the most common types. With the discovery of epidermal growth factor receptor (EGFR) and the development of gefitinib and erlotinib, which can target therapy in EGFR mutations [3], the lifetime and quality of life of adenocarcinoma patients have greatly improved. It has been reported [4] that in the NEJ 002 clinical trial Patients whose NSCLC had EGFR mutations and were treated with gefitinib showed a median progression-free survival (PFS) of 10.8 months (HR 0.30, p<0.001) and a median overall survival of 30.5 months (HR, NR, p = 0.31). These results present the following question: can EGFR mutation status be used for lung adenocarcinoma patients as a marker for treatment decisions and as a prognostic indicator? The EGFR mutation status cannot be determined for some patients because of their tumor tissue cannot be acquired, the testing method and equipment used maybe different, and quality control limitations and so on [5]; thus, timely effective treatment is not possible for those patients, and it is necessary to find efficient alternative indicators of EGFR mutation status. In the NEJ 002 clinical study [4], we found that in patients with lung adenocarcinomas positive for thyroid transcription factor-1(TTF-1) expression, the EGFR mutation rate was higher. In particular, Asians, women and nonsmokers had a much higher rate of TTF-1 positive expression and EGFR mutations. Based on this data, we hypothesize that TTF-1 expression in lung adenocarcinoma patients is correlated with EGFR mutations. The purpose of this study was to clarify whether TTF-1 expression status can be used to predict EGFR mutation status to guide clinical treatment and improve the prognosis of patients with advanced lung cancer.

## Materials and Methods

### Ethics Statement

For all of the participants, written consent was obtained after a description of the study was given and prior to conducting interviews. All of the participants signed the informed consent. This research was approved by the Shanghai Changhai Hospital ethics committee.

### Clinical Materials

We enrolled 664 lung adenocarcinoma patients in this study from May, 2010 to April, 2013 at Shanghai Changhai Hospital who had not yet received any treatment for their lung adenocarcinoma. Among the participants, 370 were male and 294 were female. The median age of the participants was 60 (24–82) years; of 664 participants,439 were nonsmokers,225 were current or former smokers;according to TNM stage,we classified stage I A in 92 cases, I B in 256 cases, II A in 52 cases, II B in 15 cases, III A in 102 cases, III B in 36 cases, IV in 111 cases. The Performance Status(PS) were showed in table 1. All patient tissues were analyzed by immunohistochemical staining to detect TTF-1 expression levels, and DNA direct sequencing was used to detect EGFR gene mutations. All clinical materials of patients had been collected from the hospitalized cases.(all public materials have been shown in table 1), Tumor stage was determined according to the 7th edition of the TNM Classification of Malignant Tumors of Union International Control Cancer(UICC), the PS was determined according to Eastern Cooperative Oncology Group (ECOG) performance status (PS).

**Table 1:** TTF-1 and EGFR mutations in lung adenocarcinomas.

Category	Subcategory	TTF-1			P	EGFR		P
		−	+−	+		Mut (−)	Mut (+)	
Age	>65 y	2	5	174	1.000	112	69	0.509
	≤65 y	8	13	458		286	193	
Sex	Male	8	14	348	0.045	253	117	<0.001
	Female	2	4	288		149	145	
Smoking history	Never	3	8	428	0.007	236	203	<0.001
	Current or former	7	10	208		166	59	
TNM Stage	IA	1	2	89	0.586	62	30	0.569
	IB	2	8	246		146	110	
	IIA	1	2	49		33	19	
	IIB	0	0	15		12	3	
	IIIA	0	2	100		62	40	
	IIIB	1	1	34		26	10	
	IV	5	3	103		61	50	
PS Scores	0	3	4	328	0.08	196	139	0.693
	1	7	12	292		194	117	
	2	0	1	13		9	5	
	3	0	1	3		3	1	

### Detection Method

#### TTF-1 Detection

The specimens were obtained from operation tissues, bronchoscopy biopsies or TBNA, CT-guided needle biopsies of lung cancers and so on. All specimens acquired were big enough, they were fixed in 10% formaldehyde and dehydrated by conventional methods. After paraffin embedding, the specimens were serial sectioned into 5–6 sections with a thickness of 4 µm each. One section was used for HE staining to verify the pathological diagnosis by two experienced pathologists. The others were used for immunohistochemical staining (Envision two-step). (The immunohistochemical Envision two step kit and the main reagent resistance TTF-1 monoclonal antibody are Denmark DAKO products). Routine slice dewaxing was performed with 3% hydrogen peroxide to remove the endogenous peroxidase, and high-temperature antigen repairing was performed. The TTF-1 immunohistochemical staining methods were in strict accordance with the Envision two-step kit instructions. After staining, the sections were observed using a light microscope.

### EGFR Mutations Detection

DNA direct sequencing: Four sites of EGFR mutations (exons 18–21) were tested. First, DNA extraction(Tris.Cl); Second,PCR amplification, PCR reaction conditions were used: degeneration at 95°C for 15 min, annealing at 65°C for30 s, and a final elongation at 72°C for 5 min.a total 35 cycles.(Exon 18 forward primer:5′ TCCAAATGAGCTGGCAAGTG 3′,Reverse primer: 5′TCCCAAACACTCAGTGAAAC 3′; Exon 19 forward primer:5′GTGCATCGCTGGTAACATCC 3′, Reverse primer:5′ TCTGGAGATGAGGGTCT 3′; Exon 20 forward primer: 5′CCGCCTGCTGGGCATCTG 3′, Reverse primer :5′GCGATCTGCACACACAGTTGAG3′;Exon 21 forward primer:5′ GCTCAGAGCCTGGCATGAAC 3′; Reverse primer: 5′ CATCCTCCCCTGCATTT 3′; EX Taq, 2×HotStart Taq PCR MasterMix, 10×Ex Taq Buffer (Mg2+ free), 10×Ex Taq Buffer,dNTP Mixture, MgCl2, ddH2O [Japan]), The amplified fragments were separated by electrophoresis using a 2% agarose gel, and the results were obtained using a gel imaging system. Third, sequencing the PCR products to forward and backward directions. A sequencing analysis map was used to determine the presence of mutations in exons 18–21 of the EGFR gene.

### Decision Criteria

#### TTF-1 decision criteria

**Figure 1:**
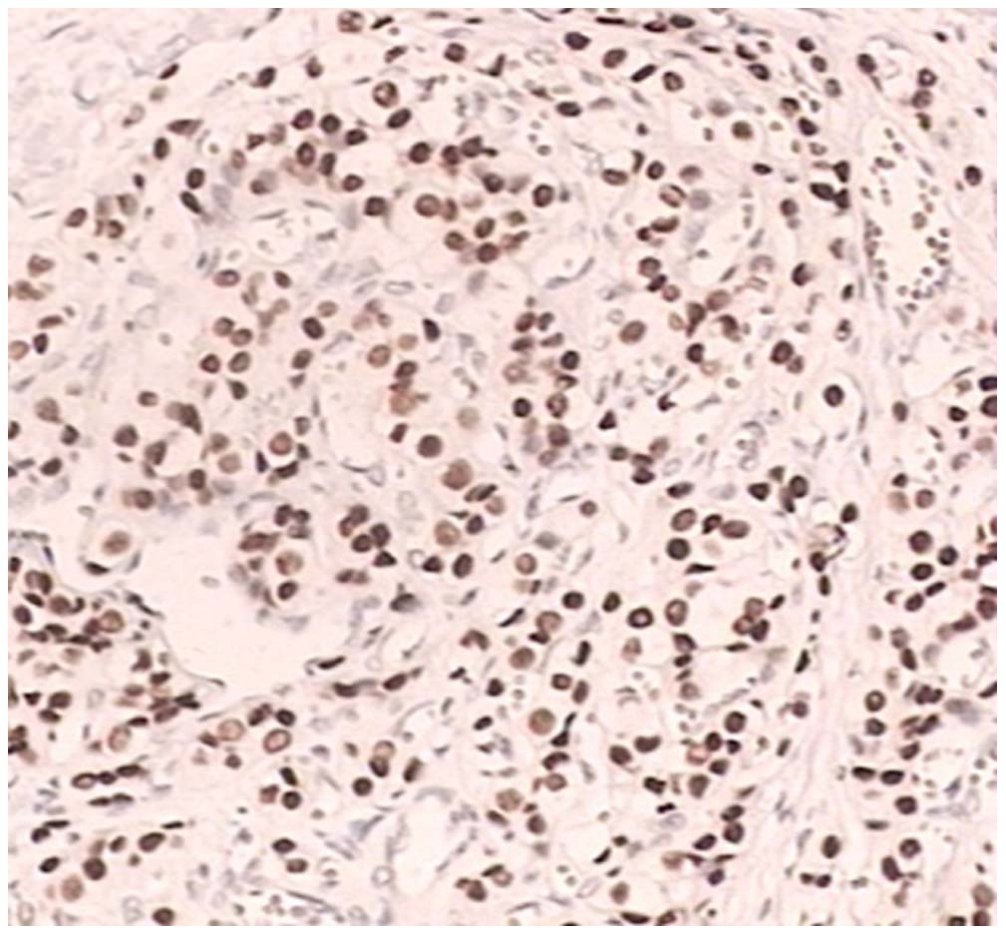
TTF-1 positive expression in adenocarcinoma cell (IHC×400).

The pathology biopsies and immunohistochemistry slides were reviewed by two experienced pathologists. The pathological diagnosis was considered definite if TTF-1 was located in the nucleus; this was indicated by tan or brown particles in the nucleus (figure 1). At high magnification 10 different views were selected; for each view, 100 tumor cells were counted. According to the percentage of positive cells, TTF-1 expression was considered − ∼+. A sample was considered negative(−) for TTF-1 expression if 0–10% positive tumor cells were observed, Partial positive (+−)if 10–50% positive tumor cells were observed, and positive (+) if more than 50% positive tumor cells were observed. The histological analysis of tumors was based on the WHO classification for cell types. [6].

#### EGFR decision criteria

EGFR mutations occur mainly in exons 18 to 21, The most commonly detected mutations are located in exon 19 (2235–2250 base deletion) and exon 21 (point mutation, 2576 T → G). The common mutation of exon 18 was G719A, exon 20 mutation was T790M(C>A), In this study, we followed the methodology of Sae-Won Han et al. [7].

### Statistical Methods

The χ^2^ test or the Fisher’s exact test were performed to assess the association between EGFR and TTF-1,which performed by SPSS 18.0 statistical analysis software. A p value<0.05 was considered important statisticaliy significance.

## Results

### Clinical Features in Patients

According to the patients clinical characteristics, we found that the TTF-1 expression and EGFR mutations were associated with gender (p = 0.045, p<0.001) and smoking (p = 0.007, p<0.001), but were not associated with patient age, TNM stage or PS scores. (1) The rate of positive TTF-1 expression (+−,+) was 97.0% for males and 99.30% for females (+−,+). The EGFR mutation rate was 31.5% for males and 51.05% for females. The TTF-1 positive expression and EGFR positive mutation rates were higher for females than males. (2) The positive (+−,+) TTF-1 expression rate was 99.08% for nonsmoking patients and 96.03% for smoking (current or former) patients. The EGFR mutation rate was 47.93% for nonsmoking patients and 25.40% for smokers. The EGFR mutation rate for nonsmoking patients was significantly higher(26.22%) than for smoking patients(46.24%), and immunohistochemical staining showed that TTF-1 positive expression was significantly higher for nonsmoking patients than smoking patients,especially nonsmoking female with TTF-1 positive expression is much higher(141/285,49.47%) (Table 1).

### EGFR Mutation

Table 2 shows that of 664 patients, 263 were EGFR mut (+) and 402 were EGFR mut (−). The mutation rate was 39.46%. The exon 19 mutation was detected in 121 cases resulting in a mut (+) rate of 18.22%. The exon 21 mutation was detected in 141 cases resulting in a mut (+) rate of 21.23%. Exon 18 and Exon 20 mutations were detected free.

**Table 2:** EGFR mutation in lung adenocarcinomas.

EGFR	Exon 19(%)		Exon 21(%)		Total
	Mut (−)	Mut (+)	Mut (−)	Mut (+)	
Mut(−)	402 (60.54)	0	402 (60.54)	0	402 (60.54)
Mut(+)	141 (21.23)	121 (18.22)	121 (18.22)	141 (21.23)	262 (39.46)
Total	543 (81.78)	121 (18.22)	523 (78.77)	141 (21.23)	664 (100)

### Comprehensive Analysis of TTF-1 Expression and EGFR Mutation Status

Using immunohistochemical method (IHC)and DNA direct sequencing method to analyze 664 tissue samples, 636 samples were TTF-1 positive (+), and 261 of those samples were EGFR mut (+) resulting in a mutation rate of 41.04%. Eighteen cases were TTF-1 partial positive (+−) and EGFR mut (−). Only 10 patients were TTF-1 negative (−), and only one of those patients had EGFR mutations resulting in a mutation rate of 10.00%. Using a Fisher’s exact test to compare TTF-1 expression rate to the EGFR mutation rate, it showed that there was a significant difference between them (Table 3).

**Table 3:** The relationship between TTF-1 and EGFR mutation in lung adenocarcinomas.

TTF-1	EGFR(%)		Total	P
	Mut(−)	Mut(+)		
−	9 (90.00)	1 (10.00)	10	<0.001
+−	18 (100)	0	18	
+	375 (58.96)	261 (41.04)	636	
Total	402 (60.54)	262 (39.46)	664	

### The Relationship between TTF-1 Expression and EGFR Mutation

Table 4 shows that TTF-1 positive(including partial positive) expression has correlation with the EGFR exon 19 mutation,but the difference is not significant (p = 0.098). TTF-1 positive expression(including partial positive) was significantly difference with the EGFR exon 21 mutation (p = 0.019).

**Table 4:** The relationship between TTF-1 expression and EGFR exon 19 and exon 21 mutations.

TTF-1	Exon 19		P	Exon 21		P
	Mut(−)	Mut(+)		Mut(−)	Mut(+)	
−	9	1	0.098	10	0	0.019
+−	18	0		18	0	
+	516	120		495	141	
Total	543	121		523	141	

## Discussion

TTF-1 is a member of the NKX2-1 family; it is expressed in the thyroid, forebrain, lung and other organs [8]. In the lungs, TTF-1 mainly exists in the type II alveolar epithelial cells and the non-ciliated bronchiolar epithelial cells. Both the enhanced surface active substances and Clara cell secretory protein region have binding sites that can maintain lung cancer cells activity [9], [10]. TTF-1 expression in lung adenocarcinoma has been thoroughly described and is considered a specific marker of lung adenocarcinoma [11]. Berghmans et al. [12 found the TTF-1 expression rate in lung adenocarcinoma to be approximately 70–88%. In our study, TTF-1 expression was negative in only 10 adenocarcinoma cases. Positive TTF-1 expression exist was found in 654 cases (positive or intensive positive) resulting in a positive rate of 98.49%. These data further illustrates that TTF-1 is a specific marker of adenocarcinoma.

EGFR is a membrane growth factor receptor that plays a role in tumor cell proliferation, adhesion, invasion and apoptosis suppression and increase angiogenesis. It plays an important role in the tumor process and patient survival and prognosis and is also closely related to chemotherapy sensitivity [13]. EGFR is a member of the type I growth factor receptor family. The EGFR gene is located in chromosome 7 in the short arm TP12-14 district. We have found that more than 90% of EGFR mutations are located in the exons 18 to 21, and the most common mutations lie in exons 19 and 21. These include the exon 19 (2235–2250) base deletion and exon 21 point mutation (2573 T replace G, L858R) [14]. Many researchers [15], [16] have found that EGFR mutations mainly occur in exons 19 and 21 and the mutant population is mainly lung adenocarcinoma patients, especially Asian women that are never smokers.

Recently, Sun et al [17] reported that TTF-1 and EGFR mutations had a correlation. Thus in our study, the analysis of the TTF-1 expression and EGFR mutations shows TTF-1 positive expression and EGFR mutations each has correlation with sex(p = 0.045;p<0.001),smoking(p = 0.007;p<0.001) in lung adenocarcinomas, especially for those female and nonsmokers, which have significant TTF-1 positive expression(Female:292/294,99.31%, Male:362/370,97.83%;No amoking :436/439,99.31%,Smoking:218/225,96.89%) and EGFR mutation(Female: 145/294,49.32%,Male: 117/370,31.62%;No smoking:203/439,46.24%;Smoking:59/225,26.22%). These results are in accordance with those previously reported by Giaccone G et al [15]. while further analyze the data, we also find the whole positive TTF-1 expression(+−∼+) is 98.49%(654/664),the female is 99.31%,the whole EGFR mutation occur is 39.46%(262/664),the occurrence rate of EGFR mutations in female is 49.32%(145/294),It is more likely occur than male(31.62%,117/370).and such is the Nonsmokers,TTF-1 positive expression of nonsmokers is more likely occur than smokers, and so it is with the EGFR mutations(Table 1, 2, 3), what is in accordance with what Sun et al and Audrey Vallee et al [18] had reported. In our hospital, exon 19 and 21 occur mutations, but the exon 18 and 20 not. The data shows exon 19 mutation account for 18.22%(121/664), exon 21 is 21.23%(141/664),the occurrence rate of exon 21 is more than exon 19(Table 2), Exon 21 mutation and TTF-1 positive expression(+–∼+) have correlation with each other, the more positive of TTF-1,the more occurrence of exon 21 of EGFR mutation(p = 0.019),it has correlation with exon 19. We further analyze the correlation of between TTF-1 and EGFR by Fisher’s exact test, it shows exon 21 mutation have correlation with TTF-1 positive expression (Table 4, 5).

EGFR mutations have now been clearly indicated as a marker of lung cancer. EGFR can be used as an independent predictive marker of prognosis. EGFR mutation testing has important significance for guiding the diagnosis, treatment and prognosis ofNSCLC. NSCLC which harbored TTF-1 positive(including partial positive) has better effective to chemotherapy. Tsao M [4] found that patients who harbored EGFR mutations showed significantly improved chemotherapy efficiency compared to EGFR wild-type patients. It is essential for EGFR mutation detection for patients with advanced lung cancer to targeted therapy. But because of the testing requirements and sensitivities of the different detection methods used and the fact that some methods for detection require more tissue samples, precise testing equipment, strict quality control of the process, more time consuming, high consumption, more people lose the opportunity for EGFR mutation detection so that they cannot get therapy timely [5]. In our study, we find the EGFR mutations was significant higher in female with TTF-1 positive expression(145/294,49.32%),especially nonsmoking female with TTF-1 positive expression is much higher(141/285,49.47%). the result is accordance with what Sun et al [17] had reported. The analysis of these patients of lung adenocarcinomas indicates that TTF-1 positive expression has correlation with EGFR mutations (p<0.001), most of all for exon 21 mutation(p = 0.019),especially for those female, nonsmokers. Kyuichi Kadota et al [19] have reported that TTF-1 correlates with tumor histologic adenocarcinoma,which is an independent predictor of recurrence for lung adenocarcinomas. Tomoya Yamaguchi, et al [20] also found that TTF-1 can induce ROR1 (tyrosine kinase orphan receptor 1) expression, which in turn sustains a favorable balance between the prosurvival PI3K-AKT and proapoptotic p38 signaling pathways; which is in part through ROR1 kinase-dependent c-Src activation as well as kinase activity-independent sustainment of the EGFR-ERBB3 association, ERBB3 phosphorylation and consequential PI3K activation.it also indicates TTF-1 expressions have correlation with EGFR, which can promote the growth of tumor cells. So both TTF-1 and EGFR can be used to diagnose and therapy for lung adenocarcinomas, because of the restriction of tumor tissues, finance, time, the status of EGFR mutations cannot be detected out timely, for we can detect TTF-1 to guide clinical therapy by immunohistochemical staining which is a simple and economic method that requires less time and manpower, So that for early therapy a reasonable plan can be carried out according to the status of TTF-1 expression. Especially for those TTF-1 negative patients who harbor less EGFR mutations can get chemotherapy timely.

## Conclusion

In conclusion, TTF-1 is not only an important cell marker of adenocarcinoma, but it also provides significant guidance for doctors to take a reasonable and timely plan for therapy with advanced lung adenocarcinoma, especially for those advanced lung cancers (Asians, female, adenocarcinoma, No smoking)who can get properly treatment timely. As markers, combine EGFR mutations with TTF-1 positive expression can guide effective clinical diagnose and therapy.
